# How does organisational culture facilitate uptake of home dialysis? An ethnographic study of kidney centres in England

**DOI:** 10.1136/bmjopen-2024-085754

**Published:** 2024-12-27

**Authors:** Kerry Allen, Karen L Shaw, Jenna L Spry, Lisa Dikomitis, David Coyle, Sarah Damery, James Fotheringham, Mark Lambie, Iestyn P Williams, Simon Davies

**Affiliations:** 1 Health Services Management Centre, University of Birmingham, Birmingham, UK; 2 Institute of Applied Health Research, University of Birmingham, Birmingham, UK; 3 Centre for Health Services Studies and Kent and Medway Medical School, University of Kent, Canterbury, UK; 4 NIHR Devices for Dignity MedTech Co-operative, Sheffield Teaching Hospitals NHS Foundation Trust, Sheffield, UK; 5 School of Health and Related Research, The University of Sheffield, Sheffield, UK; 6 School of Medicine, Keele University, Keele, UK

**Keywords:** Dialysis, Patient-Centered Care, QUALITATIVE RESEARCH, Health Equity

## Abstract

**Abstract:**

**Objective:**

The proportion of people having home dialysis for kidney disease varies considerably by treating centre, socioeconomic deprivation levels in the area and to some extent ethnicity. This study aimed to gain in-depth insights into cultural and organisational factors contributing to this variation in uptake.

**Design:**

This is the first ethnographic study of kidney centre culture to focus on home dialysis uptake. The NASSS (non-adoption, abandonment, scale-up, spread, and sustainability) framework was used to map factors that influence the use of home dialysis.

**Setting:**

We conducted focused ethnographic fieldwork in four kidney centres in England, with average or high rates of home dialysis use, selected to represent geographic, ethnic and socioeconomic diversity.

**Participants:**

Observations of patient consultations, team meetings, patient education and training sessions (n=34); and interviews with staff, patients and carers (n=72).

**Results:**

We identified three themes that can support the decision to pursue home dialysis: (a) *Encouraging patient voice and individualised support*. Kidney care teams engaged with people’s psychosocial needs and cultural contexts, and valued peer support as part of patient education; (b) *Ensuring access to home dialysis*. Transparency about all treatment options, minimisation of eligibility assumptions and awareness of inequities of access; (c) *Achieving sustained change based on benefits for patients*. This included organisational cultures which adopted quality improvement approaches and worked with wider stakeholders to shape future policy and practice.

**Conclusions:**

Willingness to pursue dialysis at home relied on patients’ and carers’ ability to place their confidence in their kidney care teams rather than how services were organised. Our study of kidney centre culture has identified approaches to patient empowerment, access to treatment and readiness for improvement and change that could be incorporated into a service delivery intervention.

**Strengths and limitations of this study**

- Identifies aspects of organisational culture that are relevant to successful home dialysis practice.
- First ethnographic study of home dialysis service delivery.
- Novel use of NASSS (non-adoption, abandonment, scale-up, spread and sustainability) framework as part of a directed qualitative analysis process.
- COVID-19 pandemic site restrictions meant the majority of interviews originally planned as face-to-face were undertaken remotely.
- The observational and reflexive nature of ethnographic data means that caution is required in interpreting cause and effect. These findings need incorporation into an evaluated service delivery intervention.

## Introduction

 Access to home dialysis varies considerably world-wide. Recently, a Kidney Disease Improving Global Outcomes Controversies Conference concluded that everyone facing dialysis should have access to home therapy.^1^ Globally, we have seen the introduction of radical clinical and government policies to incentivise home dialysis including peritoneal dialysis (PD)-first, PD-favoured and home dialysis-first.^2^ In the UK, current National Institute for Health and Care Excellence (NICE) guidance recommends that choice of dialysis modality, including whether this is at home or within-centre, should be based on the preferences of patients and their families.^3^ However, there is significant variation in the use of home dialysis across kidney centres in the UK. The uptake can be as low as six and as high as 29% of the prevalent dialysis population.^4^ There is also evidence of sociocultural inequity of access to home dialysis. Those living in areas of higher deprivation and/or those belonging to Black, Asian and mixed race minority ethnic groups are especially under-represented.^4^,^6^ In the UK context, ethnicity is categorised as per the Office of National Statistics in Renal Registry and key national level kidney care analyses.^4 5^ These disparities suggest that there are barriers to home dialysis access within centres, that are likely to be complex, and may include wider institutional policy, unconscious bias, lack of staff education and variable investment in home dialysis-related innovations.^7^,^9^

Indeed, a growing body of evidence identifies the factors that facilitate and impede successful uptake of home dialysis. These include the importance of early and individually tailored patient education, staff education about home dialysis options, technological innovation, psychosocial and peer support.^10^,^14^ Healthcare professional factors associated with lower home dialysis uptake include fears and concerns, working style and communication skills. At the healthcare system level, the presence of competing treatments, financial incentives for providers favouring dialysis within-centres and lack of space at centres for home dialysis training are important.^15 16^ The evidence often highlights a practice-theory gap between the logic of interventions intended to ensure equitable access, such as shared-decision-making, and their implementation in practice;^10^ suggesting further research is needed to understand this.^17^ Typically, current evidence does not specifically address organisational culture, as distinct from how kidney failure services are organised. Exploring these barriers through the lens of kidney centre culture can offer valuable insights about how to close this gap and achieve the intended benefits of home dialysis interventions.

Organisational culture is conceptualised as shared ways of thinking that drive behaviour and influence performance.^18^ In the healthcare sector, organisational cultures are characterised by their complexity. The presence of multiple subcultures, different governance structures and variable access to material resource all make the links between culture and outcomes less straightforward.^19^ This ethnography was part of Inter-CEPt; a large mixed-method study that aimed to explain unwarranted centre variation in home dialysis uptake and develop a service delivery intervention to address it.^20^

## Methods

In our comparative ethnographic approach, four kidney centres each constitute a ‘case study’ site, considered sufficient to generate new insights.^21^ The ethnographic study aimed to provide an understanding of the interplay between health professionals, patients and carers, the kidney centre and its culture^22^ and to identify contextual factors that can support the decision by patients and their families to pursue home dialysis. We have followed the ‘Consolidated criteria for reporting qualitative research’ (COREQ) guidelines.^23^ Ethical approval was obtained from the Wales Research Ethics Committee (ref. 20/WA/0249) on 18 September 2020. Informed consent to participate in the ethnographic study was provided by all participants via a consent form and agreement to participate. All data collection was undertaken by three research team members KA (PhD, Medical Sociologist, Associate Professor, female), KLS (PhD, Psychologist, Research Fellow, female) and JF (BNurs, Research Fellow, female). These ethnographers had NHS ‘research passports’ with each site to allow them access to undertake the research, including ‘good clinical practice’ training.

### Sample: site and participant selection

We used a positive deviance approach^24^ to sample the four sites in England, with the intention of identifying practices that facilitate home dialysis uptake. Positive deviance approaches are increasingly used in qualitative health services research to identify feasible solutions that are currently underway.^25^ We focused on four kidney centres that demonstrate either above the median or high home dialysis uptake rates, expressed as the proportion of the whole renal replacement population (including transplantation), using Renal Registry Data from 2018. Selection of these four case study sites was designed to observe successful practice, while maximising diversity and involved two-stage purposive sampling.

At stage one, each kidney centre in England was assigned to relevant categories based on a home dialysis uptake taxonomy developed using UK Renal Registry data:^26^ (1) high uptake of home dialysis (top 10% nationally); (2) high uptake of home dialysis among ethnic minorities (top 10%); (3) home dialysis uptake for all patients around the national median and (4) home dialysis uptake for ethnic minorities around the national median. At stage two, a single case study site was selected from each of these four categories. The final decision on which centres to include was made to ensure a balanced selection based on maximum geographical variation; sociodemographic characteristics of a centre’s patient population (less affluent/affluent/mixed); ethnic diversity (high/low ethnic minority populations) and equal representation of centres with and without transplantation on site. Table 1 displays the home dialysis prevalence at the sites.

**Table 1:** Site characteristics: home dialysis prevalence for all patients and ethnic minority patients, by site

Case study site	Site 1	Site 2	Site 3	Site 4
Proportion (%) on home dialysis (PD or home HD)*	23	13	9.3	9
Home dialysis uptake in top 10% of kidney centres, all patients	X	X		
Home dialysis uptake between median and top 10%, all patients			X	X
Proportion of ethnic minority patients using home dialysis (%)	31	15.5	16.3	15
Home dialysis uptake for ethnic minority patients in top 10% of kidney centres	X			
Home dialysis uptake for ethnic minority patients between median and top 10%		X	X	X

* aAs a proportion of the whole population on kidney replacement treatment, that is, all dialysis and transplant patients. At the time of site selection, the median national proportion on Hhome Ddialysis was 7.6%.

HDhome dialysisPDperitoneal dialysis

The ethnographers did not have a previous relationship with the study sites. A 1 month site set-up period in each participating centre allowed researchers to consult with stakeholders prior to the start of data collection and to gain familiarity with the working procedures within each centre. This codesign phase of the ethnographic fieldwork included conversations with clinical leads, key staff members and patient representatives. The researchers explored how services were organised/experienced and the areas of the service they see as relevant to the study.

Sampling of patient participants and their carers was purposive. As our Inter-CEPt study is primarily concerned with initial treatment decision-making, we sampled participants who were anticipated to start dialysis within the next 3–6 months. Identification of this sample was guided by the clinical judgement of the staff at each site and discussed during the site preparation phase. This included ‘emergency’ patients who presented with an immediate need for dialysis and those for whom a longer term decision regarding preferred modality was not yet made. Patients starting dialysis following a failed kidney transplant were included.

Patients and carers were first approached about participating in the study by the centre staff (consultants or nurses). They were given a participant information sheet which introduced the researchers, described the aims of the study and what their participation would involve. In instances where patients and carers were interested in being involved in the study, sites collected consent and passed the details of participants on to the research team. No members of the research team had particular biases or personal reasons for involvement in the topic, thus none were reported to participants.

Table 2 displays the characteristics for participants interviewed or observed in consultations. Not all patients who were observed also took part in interviews, this explains the difference between the number of patient interviewees (n=24) and patients participating overall (n=36). Five patients who consented did not go on to be interviewed, due to lack of availability. These patients are not included in table 2.

**Table 2:** Participant characteristics: patients and carers

Characteristics	Categories: number
**Patients/carers**	Patients: 36Carers: 7
Age	40–49: 550–59: 1560–69: 670–79: 1380–89: 4
Gender	Female: 22Male: 21
Ethnicity	Asian (British or mixed Indian): 3Black (British or mixed African, Caribbean): 6White (British or European): 30Unspecified: 4
Multiple index of deprivation, quartile* (1=most deprived, 4=most affluent)	Quartile 1: 11Quartile 2: 21Quartile 3: 5Quartile 4: 6
Planned or emergency route to treatment	Planned: 38Emergency: 5
Total patient and carer participants	43

* Score based on patients’ home postcode to identify the level of deprivation of the area they live in, using the ranking system of the IMD (every area in England is ranked from 1=most deprived, to 32 844=least deprived), and converting this rank into deprivation quartile.

IMDIndex of Multiple Deprivation

Staff participants included those who had regular contact with patients concerning their treatment, or who have oversight of decision-making processes within the centre. The participant job titles are listed in table 3.

**Table 3:** Number of staff participants by job title

Job title	N
Consultant nephrologist	12
Senior registrar	1
Nurses	
Home therapies nurse specialist	2
Home therapies lead nurse	1
Lead for shared care	1
PD senior clinical nurse specialist	1
PD nurse	2
Home therapies trainer	2
Low clearance nurse specialist	5
Lead nurse	1
Nurse manager	1
Nurse consultant	1
Specialist nurse	3
Support assistant nurse	1
Allied health professionals	
Dialysis technician	1
Home therapies dietician lead	2
Social worker	1
Young adult support worker	1
Clinical psychologist	1
Counsellor	1
Total staff participants	41

PDperitoneal dialysis

### Observation

We conducted observation in the following settings: in-patient and out-patient areas, including reception areas, waiting rooms, wards (n=4); individual consultations and patient education (n=26); online group patient education (n=2); home dialysis training (n=1) and a multidisciplinary team meeting (n=1). Researchers took fieldnotes during and after observation, which were discussed weekly by the research team. Fieldnotes are viewed as an essential component of rigorous ethnographic approaches and are a standard criteria for qualitative research reporting.^27^

Individual consent was gained when observing consultations, including patients, accompanying carers and health professionals—as all were considered participants in their own right. For all other observations, people were informed about the research via posters or by their trainers. In line with the approved ethical approach, we did not record individual consent, or capture any personal or identifiable details in these more general observation settings.

### Semistructured interviews

We interviewed 41 staff, 24 patients and seven carers. Restrictions at sites due to the COVID-19 pandemic during 2021–2022 meant that researchers did not gain access to sites for the initial year of fieldwork. For this reason, the majority of interviews that had originally been planned as face-to-face were undertaken on by video or audio call. All staff interviews, and all but three patient or carer interviews, were undertaken during the period of COVID-19 public health restrictions, by video or audio call. The three in-person patient or carer interviews were conducted in a private room at one of the study sites. Patients and carers received separate interviews in order to distinguish their unique perspective. All interviews lasted between 20 and 45 min.

Interviews explored how patients were making treatment choices and the factors that influenced these choices, including but not limited to home dialysis. Staff interviews focused on the approach of centres to increase the uptake of home dialysis and identified relevant documentary data, such as organisational policies, strategies and materials produced for patient counselling and education.

All participants were sent a copy of the interview topic guide prior to the interviews. A public and patient advisory panel codesigned the interview topic guides, as well as all patient/carer targeted materials (participant information and consent forms). Suggestions in changes to terminology were adopted. Topic guides for staff were piloted and discussed with the clinical leads at each site in the preparatory month ahead of data collection. All interviews were designed as a stand-alone data collection point and interviews were not repeated with any participants. All interviews were recorded on encrypted devices and transcribed verbatim.

### Analysis

Fieldnotes, interview transcripts and documentary evidence were imported into the qualitative data-analysis software QSR NVivo V.12 for thematic analysis. Case sites were initially analysed separately to capture site-specific effects. Decisions about when we had reached data saturation were also taken per site, as well as across the study. We operationalised the process of data saturation as the point when new data collected produced little or no change to the codes.^28^

Our analysis is guided by seven domains:

1. Condition/illness (kidney failure)
2. Technology (ie, home dialysis)
3. Value proposition (to both the patient and the provider)
4. Adopters (staff, patients and carers; and their role, identity and the input required of them).
5. Organisation (including values, capacity, capability and readiness to change)
6. Wider system (including policy context, legal issues, sociocultural context).
7. Embedding and adapting the technology over time.

This conceptual framework, NASSS (non-adoption, abandonment, scale-up, spread and sustainability),^29^ was designed to enable nuanced understandings of complex service delivery goals and the factors that aid or inhibit their progress. We followed Assarroudi *et al*’s^30^ ‘directed’ qualitative analysis process where existing theoretical frameworks (ie, the NASSS conceptual framework) led our initial analysis, while allowing themes outside of this framework to be generated. The use of the NASSS framework^31^ enabled a conceptual mapping of themes, which influenced the uptake of home dialysis, and how these factors interact.

A multidisciplinary team of researchers undertook coding and analysis. The ethnographers, KA, KLS and JLS, coded the majority of data that they had collected. Weekly team meetings refined the interpretation of data as a measure to improve rigour and reduce bias. Throughout the analysis, data excerpts from each site were coded independently by different members of the team and subsequently compared with quality assure consistency of approach. The wider multidisciplinary research team consisted of LD (PhD, Anthropology, Professor, female), IPW (PhD, Policy and Management, Professor, male), SaD (PhD, Applied Health Research, Research Fellow, female) and DC (Patient and Public Involvement coinvestigator, male). The patient and public advisory group provided feedback on the development of findings at a preliminary point and when findings were close to being finalised.

### Patient and public involvement statement

The study involved people with lived experience of kidney care at all stages. A patient coapplicant (DC) was a core member of the ethnographic study and joined monthly meetings to manage research design, data collection, analysis and address any fieldwork challenges. This coapplicant chaired a patient and carer advisory group which represented people from areas of high socioeconomic deprivation and from ethnic minority communities. The ethnographers ran four workshops with the patient advisory group before and during the ethnographic study: (1) to codesign patient and carer materials and gain insights into what aspects of the study the group felt were most important; (2) to discuss recruitment strategies and how to maximise participation; (3) to gain feedback on emerging findings and (4) to finalise the ethnographic results and think through the dissemination messages that will have the most positive impact on people with kidney disease, their families and communities.

## Results

We observed a variety of approaches to organising services across the four sites. There did not seem to be a clear pattern or model that ensured successful home dialysis uptake. We identified three overarching themes describing the features of centre culture relevant to home dialysis use. These are described in table 4 and mapped against the NASSS domains to indicate how subthemes link to centre practice and wider care systems.

**Table 4:** Key themes and NASSS (non-adoption, abandonment, scale-up, spread and sustainability) mapping: a framework for home dialysis activity

Themes	NASSS domains
*Encouragingpatientvoice and individualised support.*Teams humanised their healthcare approaches by recognising the impact of kidney failure on each person’s life. There was engagement with non-medical aspects of treatment decisions, such as psychosocial and cultural needs, and value peer support as part of patient education.	*Value proposition*—patient education explored how home dialysis offers value and fits individual’s lifestyles.*Adopters*—staff communication/treatment decision approaches that maximised patient and carer input in treatment decisions. Staff recognised and attempted to address barriers faced by ethnic minority patients or people with lower socioeconomic circumstances.*Organisation*—nursing staff capacity and capabilities to support high levels of patient education and individualised support.*Wider system*—inclusion of peer, psychological and social support can be influenced by policy and capacity outside the centre.
*Ensuring access to home dialysis*Transparency about all treatment options, minimising assumptions about eligibility, working with people to overcome perceived barriers.	*Condition*—thorough assessment to ascertain real, not presumed (in)eligibility based on medical history, comorbidities or social circumstances.*Technologies*—home dialysis machine choices that considered ease of training, use and space. Centre initiatives that increase access and eligibility for example, assisted PD.*Adopters*—staff knowledge and patient education skills. Support for home dialysis that reassured the patient at the point of decision-making for example, dedicated training team, home assessment and set up of machines, 24-hour support line.*Organisation*—local policy and pathways designed to facilitate early consideration of treatment types and time to overcome barriers for example, housing/deliveries or supporting patients with fear of self-needling via ‘shared care’ pathways.*Wider system*—local authority collaboration to resolve practical housing issues that can limit access.
*Achieving sustained change based on benefits of home dialysis forpatients*.The long-term mission for sustained change is motivated by a visible belief in the benefits of home dialysis for patients. This was seen in the influence of effective leadership, improvement and learning built into routine working and outward facing work with the public, in regional and national networks and charities.	*Technologies*—investment in home dialysis technology and service innovations for example, ‘shared care’, home HD machines.*Value proposition*—widespread staff belief that the quality of life and clinical outcomes warrant the investment of organisational resource in home dialysis.*Adopters*—staff and patients promote home dialysis and enhance knowledge and attitudes across the organisation. Peer supporters have a role in empowerment and education of patients/carers.*Organisation*—culture of improvement and learning around home dialysis with regular opportunities for staff to reflect collectively on successful practice.*Wider system*—patients and staff were engaged with wider charities, regional networks and working towards an awareness of the benefits of home dialysis outside the kidney centre.*Embedding over time*—kidney centres were involved in developing home dialysis policy and influencing decision-making. Senior staff encourage coproduction of services with home dialysis patients.

HDhome dialysisNASSSnon-adoption, abandonment, scale-up, spread and sustainabilityPDperitoneal dialysis

### Theme 1: encouraging patient voice and individualised support

People approaching kidney failure described the scale of emotional and practical upheaval that they were processing. We observed that when kidney centre staff showed an appreciation of people’s distress and changing self-image, they felt able to develop effective patient education. This organisational value was marked by communication skills which encourage patients to raise their unique concerns and revisit information gradually over time. Fieldnotes from a patient education session capture the qualities of the facilitator. Ethnographers observed the perceived importance of tone during interaction and how patients were encouraged to engage in decision-making.

The group leader strikes me as gentle and calming and greets people by name…He recurrently emphasises core aspects of patient-centred care:

- *Patient agency—they can take an active role.*
- *He often invites patients to raise concerns with him or any member of their team.*
- *Shared decision-making and patient individuality. Team will work to ‘personalise’ care, but it’s ‘your decision’(Fieldnotes from patient education observation, ID_S1O15*).

Inclusion of non-medical support in patient education was a feature of individualised approaches. All sites prioritised psychosocial support through their consultation styles, training and information events, and worked with psychologists, social workers and peer supporters. National charity-based materials promoting additional support were accessible in waiting area displays, as well as being directly distributed during consultations. The importance of the family context for treatment decisions was acknowledged, with carers routinely invited to appointments. This patient describes the value of including non-medical support, in terms of the way social work input had helped them to understand and start claiming benefit entitlements:

They definitely got me through some really tough times. And the support there was, like social workers were rather helpful with like forms, when you’ve got to talk to people on the phone and you might not be confident. I had somebody there that if I got stuck she could take over. (Patient, ID_P307)

Kidney centre staff encouraged patients and carers to voice their unique questions and concerns, as a means of understanding sociocultural inequalities. We observed care teams’ attempts to address barriers such as housing, self-efficacy and stigma of illness. Cultural and socioeconomic barriers were identified, in part because people in senior roles encouraged staff to take an exploratory approach, ask questions and listen to how people feel about treatment options.

I say this to our trainees when they come on placement, it’s okay to not know about somebody’s cultural background, but it’s not okay to not ask. So on choosing a dialysis, explicitly ask them about cultural beliefs, about their community, about their religion. (Consultant nephrologist, ID_S211)

Staff involved in treatment decision-making conversations with patients described how economic, cultural and psychological barriers are often interlinked, or experienced as ‘intersectional’, by individual patients and their families. In the following example, a consultant spoke about the importance of addressing housing and social work referrals as early as possible, in order to address structural issues such as income and living conditions. They felt that a certain level of material security was required before people could build confidence in their ability to dialyse at home.

For people that have quite challenging lives, living in social deprivation, their sense of self-belief is really low. The difficulty we find is that you can’t build self-efficacy when all the challenges of social deprivation and difficulty still exist. If somebody’s not got a house to live in, then that is their priority need, and until you can address those things, it’s going to be very hard for them to build their confidence, because there will constantly be all those other factors that undermine it. (Consultant, ID-S304)

### Theme 2: ensuring access to home dialysis

Home dialysis choices were underpinned by an emphasis on ‘finding the right treatments for the right people’ (Nurse, ID_S111), rather than increasing home dialysis uptake per se. Observation of consultations revealed that fully exploring eligibility and performing assessment of suitability for treatments was routine practice. In this way, clinical staff ensured that no assumptions were made based on patients’ medical records that could unduly rule out home dialysis for example, cardiovascular disease, cognitive function or peritoneal damage (*Observation fieldnotes from patient consultations, ID_S3O3, S2O6*). There was similar effort to avoid assumptions about non-clinical circumstances that might limit eligibility, such as the need for a carer for home haemodialysis or when people had little space at home.

…those potential issues, we can overcome them. Storage spaces or lack of help for somebody, can we overcome all those things? And if so, we would then discuss all that with the patient, if that was the modality they wanted. (Nurse, ID_S122)

Facilitating access to home dialysis meant organisations prioritising initiatives, technologies and staff roles that support the transition from hospital to home and that offered patients the reassurance they needed as they made treatment decisions. Shared care approaches offered patients the opportunity to make decisions about treatment location in their own time by supporting them to develop their haemodialysis self-management skills. Shared care staff worked with patients to overcome fears about self-needling and to train them to use machines selected for simplicity of use. Home trainers, technicians and dedicated support lines were available to oversee the start of home dialysis programmes and provide ongoing support. In the case of home haemodialysis, one home trainer described how their focus is to keep the machine set up as uncomplicated as possible for the patient.

I also am involved in the home assessment process to make sure we’ve got suitable storage and water connections/drainage connections for our machines. Once we get the patients trained, it’s my job to connect those machines up so the patients are ready to go, so we don’t need any other people involved. (Home trainer, ID S436)

A related finding was that access to home dialysis also depended on the cultural characteristics and language skills of the team. Sites acknowledged that these factors influence patient decisions and the quality of the service they receive.

One of the most important things within any dialysis programme is that our staff reflect our population. And I am ever so grateful to my team, and we are a very varied multicultural team and even in regard to having PD nurses that speak different languages so that we can communicate with our patients. (Nurse, ID_S303)

### Theme 3: sustained change based on benefits for patients

All sites shared a strong commitment to optimising home dialysis care and uptake, expressed in terms of the benefits for patients. By contrast, cost benefits, performance targets and financial incentives for providers were viewed as weaker drivers for motivating staff. The sites were not partisan towards home dialysis in their patient training, rather they were able to articulate the benefits of all dialysis types in terms of benefits for patients, rather than defaulting to in-centre options.

The inclusion of patient leaders and their role in the coproduction of services allowed the benefits for patients to be understood first-hand. We observed patient expertise and positivity for home dialysis being harnessed in training sessions codelivered with patients and in other peer-support initiatives. Patients with experience of home dialysis were included as a valued part of service delivery, offering opportunities for those new to dialysis to see how treatment can be incorporated into everyday life. Many patient leaders were also part of kidney care networks, research and quality improvement projects, providing patient perspectives beyond the centre, at regional and national levels.

All sites shared a focus on developing internal cultures of quality improvement and innovation. For instance, ‘Advanced Kidney Care’ team meetings provided space for reflection and sharing practice. We also observed effective communication and mutual respect between modalities. An example of this was PD and home haemodialysis teams working on a transition pathway, avoiding a period of in-centre haemodialysis. Staff talked in general terms about the positive aspects of their organisational leadership and culture as the ability to innovate, take contained risks, an openness to new ideas from the team and clear articulation of the goals of the centre.

I’d say there’s a culture of openness. They’ve [senior leadership team] got quite high standards, in a nice way, and so I think it is the combination of having people that are friendly, open, and listening, but also willing to hold people to high standards and follow up when that’s not been done. And quite innovative thinkers. (Consultant Nephrologist, ID_S115)

A commitment to sustained progress in home dialysis was evident in high levels of external engagement with networks, commissioners, other hospitals and trusts, charities, research, healthcare companies and local communities. Staff belonged to groups which sought to influence policy and practice at regional, national and international levels. Other specific benefits of collaboration were seen as follows: more efficient working, for instance through workforce planning or developing business cases; raising public awareness and profile of home dialysis and gaining access to medical products and training. Participants suggested this outward facing approach was the way to plug gaps where kidney centres were undersupported or duplicating effort. This quote from an interview with a nephrologist gives an example of issues he has raised with wider stakeholders to ensure local problems are understood. In this case, how national benchmarks about quantity and type of staff required for home dialysis could help with local planning.

Having a system wide approach of what’s necessary is helpful. If there were some really good quality, nationally endorsed work around what is the kind of minimum workforce required for PD and home haemodialysis, then you could start to benchmark for yourself. (Consultant Nephrologist, ID_ S340)

## Discussion

Through our ethnographic case studies, we identified three approaches that characterise how organisational culture can have a positive influence on home dialysis uptake: (1) encouraging the patient voice and individualised support; (2) ensuring access to home dialysis and (3) achieving sustained change based on benefits for patients. Our findings suggest several organisational priorities that have fostered patient confidence in home dialysis. Centres seeking to increase their home dialysis use should consider the extent to which all aspects of their practice will reflect these principles.

Patients and carers responded positively to instances where their individual circumstances had been explored, heard and acted on. The broader implication for practice is that patient education styles are enhanced by adopting flexible approaches that respond to patient priorities and appropriate paces of delivery. Engagement with non-medical aspects of care were essential in preparation for home dialysis, including psychosocial support needs. Peer support and the ability to link with social benefits and housing issues were important areas of reassurance for many considering home dialysis.

Success in home dialysis was dependant on issues of ‘access’ being central to service design. Access was considered as part of organisational decisions about how pathways are envisaged; how centres are staffed, including representation of ethnic minorities and which technologies and treatment options are available. Assisted PD and supported self-management of haemodialysis on wards were specific examples of investments which had helped alleviate patient fears about home dialysis. This ethnographic study identified how kidney centres used their ‘service improvement’ projects and external partnerships to enhance knowledge of patient benefits of home dialysis.

Our findings suggest that people’s decisions to use home dialysis are well supported when interactions with patients are carefully considered, and when the core ‘patient experience’ mission of the service is understood by all involved. Relational and value-based leadership approaches have regained currency as promising postpandemic healthcare leadership styles.^32 33^ Clinically-led, value-based approaches articulate the core ethical and moral purpose of services and set out expectations for patient experience and outcomes.^34^ These have been viewed as being particularly advantageous as the global health sector responds to social change, widening inequalities, limitations on resources, decreased morale, and rapid technological development.

Ethnographic studies about kidney centres have been limited and to date have not targeted home dialysis, focussing more broadly on paediatric settings^35 36^ and haemodialysis units.^37^ This ‘focused ethnography’ is novel in its approach, highlighting aspects of organisational culture that are relevant to successful home dialysis practice, particularly concerning presentation of treatment choice and decision-making, for example by adopting the stance of ‘presumption of eligibility’.^38^ Where previous studies have identified barriers and promising interventions, our findings also focus on the underlying organisational values which can assist the effective implementation of home dialysis innovation. These findings offer insights for leaders and managers directly responsible for shaping the organisational culture of kidney centres. It also informed the design of the quantitative components of the Inter-CEPt study such as the National Survey of Home Dialysis Centres in England by ensuring that questions related to organisational culture were included. The results of this survey corroborated the ethnography findings, suggesting that the findings are indeed generalisable to the UK National Health Service.^39^

This is the first time that the NASSS framework has been applied to the adoption of home dialysis. Our primary aim in mapping to the NASSS domains was to create a framework for future activity that might be implemented to support centres in expanding their use of home dialysis. It has helped us to identify key areas, for example within staff training, which should be applied across kidney services where there may be unconscious bias about eligibility for treatment. It has highlighted the need for a stronger engagement with the technologies for home dialysis and a more transparent approach to their use. It has also underlined the importance of centres being outward looking, engaging with industry, national and international policy, and quality improvement.

### Strengths and limitations

Our study has a number of limitations. Our fieldwork was conducted during 2021–2022, largely during the COVID-19 pandemic. The team faced challenges such as changes in research contacts at sites due to the deprioritisation of non-COVID-19 research typical during this period.^40^ During our first year of fieldwork, the in-person contact we had planned was conducted remotely, including site visits, training for recruiters and all data collection. Although this was not an intended design, the online data-collection methods meant we could more accurately reflect the service delivery at that point in time for example, online patient education and telephone consultations. We conducted interviews as remote audio-visual meetings or by telephone. Towards the end of the fieldwork period, we were able to gain in-person access to sites to observe training, patient education, consultations and conduct three patient interviews. We found it difficult to recruit patients and carers. Although our final sample of interviewees was smaller than initially intended, we were able to reach data saturation, specifically measured as the point at which no new themes were identified in three consecutive interviews. A patient advisory group provided additional input and reflection on patient and carer findings at four points in the ethnographic study.

Our research was undertaken in four sites across England, selected because of their relatively strong performance in offering home therapies. We did not include poorly performing sites partly because it was unlikely that they would inform best practice and partly because they may be reluctant to participate. A limitation to generalisability is that all these sites are working within a healthcare system that is free at the point of care and staff are salaried independent of the treatment modality patients choose. This might explain why clinicians reported giving a low priority to financial considerations, preferring to frame their treatment goals in terms of the benefits for patients.

## Conclusion

Aspects of organisational culture contribute to fostering the confidence required for patients to make home dialysis choices. While each kidney centre has a unique organisational culture, shared values associated with successful home dialysis uptake were identified as follows: a focus on encouraging individual patient perspective and tailored responses; optimising access to home dialysis and a guiding belief in the benefits of home dialysis for patients. These values underpin practices that support patients to make the dialysis choices most suited to their personal context, including home dialysis.

## Data Availability

Data are available upon reasonable request.
