# Supplementary material for: How does organisational culture facilitate uptake of home dialysis? An ethnographic study of kidney centres in England
Source: BMJ Open. 2024 Dec 27;14(12):e085754. doi: 10.1136/bmjopen-2024-085754 (PMC11683977; doi:10.1136/bmjopen-2024-085754)
Supplement: Supplementary file 1 [file bmjopen-2024-085754.reviewer_comments.pdf]

PEER REVIEW HISTORY

BMJ Open publishes all reviews undertaken for accepted manuscripts. Reviewers are asked to complete a checklist review form and are provided with free text boxes to elaborate on their assessment. These free text comments are reproduced below.

ARTICLE DETAILS

Title (Provisional)

How does organisational culture facilitate uptake of home dialysis? An ethnographic study of kidney centres in England

Authors

Allen, Kerry; Shaw, Karen L; Spry, Jenna L; Dikomitis, Lisa; Coyle, David; Damery, Sarah; Fotheringham, James; Lambie, Mark; Williams, IP; Davies, Simon

---

VERSION 1 - REVIEW

**Reviewer**	**1**
**Name**	**Pauly, Robert**
**Affiliation**	**University of Alberta**
**Date**	**24-Apr-2024**
**COI**	**None**

Thank you for allowing me the opportunity to review the manuscript submitted by Allen et al. entitled "How does organizational culture facilitate uptake of home dialysis? An enthnographic study of kidney centres in England."

Firstly, allow me to declare that I have no experience with ethnography and can provide no comment on the methodological merits of this submission. My comments should be viewed in this light and may not be as relevant because of this. I can only provide very high level comments based on my expertise in overseeing and delivering home therapies in a sizable single centre home dialysis program of over 400 peritoneal dialysis and home hemodialysis patients where home dialysis makes up ~30% of prevalent ESKD patients.

Allen et al. describe a qualitative study where they seek to understand elements of organizational culture that presumably contribute to centre variation in home dialysis uptake. The team interviewed relevant patients, care partners, health professionals and administrators in order to identify themes that facilitate patients and their partners to select home dialysis. The research team focused on 4 kidney care centres with high or average uptake of home therapies and inclusive of a high or average population of ethnic minorities performing home dialysis.

Fieldnotes, interview transcripts, and physical material (presumably pamphlets, educational material, etc) were imported into specialized software for thematic analysis until saturation. Themes were developed using a specific conceptual framework (NASSS), with which I am, unfortunately, not familiar.

Three broad themes of organizational culture emerged from this study as supportive of home therapies uptake: (a) uptake is associated with an individualized (patient-centric) approach to modality selection, (b) uptake is associated with providing access to those modalities when emphasis is placed on overcoming cultural/financial/logistical barriers to selecting home therapies, and (c) uptake is associated with a home program that is innovative in remaining patient-centred.

Major concerns:

1. I have some concerns with the novelty of the conclusions presented. Does it really surprise anyone that programs which are patient-centred, offer a menu of therapy options and have an administration that openly advocates for home therapies would, in turn, have higher uptake of home therapies? This is the subject of multiple narrative and systematic reviews over the decades. Is the novelty in that this is an ethnographic study? If so, is this simply using a different research approach to identify issues that are otherwise either self-evident or already known? To turn this around, wouldn't it be a surprise if one were NOT to have identified "transparency about all treatment options, minimizing assumptions about eligibility, working with people to overcome perceived barriers" as facilitators of home therapies uptake? Or more bluntly, to identify that limited transparency about treatment options were to lead to increased uptake? I do think that the authors would benefit from clearly articulating what about their approach and conclusions crystalizes something new.
2. The choice of centres to include: I find it curious to have included centres that only had a 9% or 13% prevalence proportion of home therapies rather than focusing on the one centre that has 23% uptake. The former sites would in many jurisdictions be considered poor performing sites and an interesting question might have been to explore the differences in the high performing site compared to those with less uptake. What makes the 23% site so much more successful than all the others? This study doesn't somehow stratify their results so see what the differences are between good sites and average sites. Are there elements to the three themes they have identified that are **different** in the 23% site versus the other three which are so clearly discordant? The way the study is framed now, there would be nothing unique to learn from the one site that actually does a very good job at attracting home therapies patients. This study hasn't leveraged the fact that one site is so much better than all the others to learn what that site does that is unique and makes them so successful. What are those characteristics? As it is, we are learning from average sites and mixing the findings with that of a good site? I am much more interested in what site 1 has to teach us about their culture compared to other institutional cultures – I wouldn't know that on the basis of the present results.
3. The methods section makes a point to comment on the inclusion of sites with higher than typical uptake of home therapies among ethnic minorities but then this issue doesn't come up again at all. Was this intentional? Was there something to be learned specifically about programs with high ethnic diversity? If so, this doesn't

come out at all later. If it doesn't matter, then why was this selection criteria important? Related to point (2) above, is there something about the culture of site 1 with respect to recruitment of ethnic minorities that allows this site to have twice the prevalence proportion of ethnic minorities compared to sites 2-4.

Minor concerns:

1. Page 8 lines 13-29: I am unclear who are what is meant by "non-participant observation" – is this meant to describe review of physical locations (waiting rooms, clinical spaces, training areas, etc), promotional and educational material, etc? This is confusing because the section ends by stating that individual consent was obtained and that all involved were considered "participants in their own right."
2. I find theme 3 to have a confusing title and isn't intuitive relative to the content of the paragraphs that follow or to the material presented in the table 4.

**Reviewer**	**2**
**Name**	**Ethier , Isabelle**
**Affiliation**	**Université de Montréal**
**Date**	**25-Apr-2024**
**COI**	**None to declare**

I read with interest the manuscript "How does organisational culture facilitate uptake of home dialysis? An ethnographic study of kidney centres in England" by Kerry Allen and colleagues. This study provides interesting findings on certain aspects of kidney centre culture having an impact on the uptake of home dialysis in the UK.

The manuscript is well written and relevant to an international audience. The involvement of people with lived experience of kidney care at all stages of the study is also very interesting and well highlighted.

I have provided some comments/questions for clarifications and potential improvements to the manuscript.

1. The abstract very well describes the aim of the study. However, the present study is only very briefly mentioned in the Introduction section and the aim is not mentioned clearly in the main text. The objective of the study should be added at the end of the introduction.

2. "Sample : Site and participant selection" subsection – last sentence of 1st paragraph: "Selection of these four case study sites was designed to observe successful practice whilst maximising diversity, involved two-stage purposive sampling." – The authors might wish to add the word "and" before the word "involved" or modify "involved" for "involving".

3. The authors discuss variability in uptake of home dialysis related to ethnicity in the introduction and designed their study to include centers with high uptake of home dialysis amongst ethnic minorities. Ethnicity of the participants is also described in Table 2. However,

what “ethnic minorities” represent in the setting of the study is not described. Since ethnic minorities vary widely across regions/countries, it would be helpful to the readers to briefly describe the UK population and what constitutes “ethnic minorities”.

4. The sentences referring to Tables 2 and 3, as well as the first sentence from the “Semi-structured Interviews” subsection should maybe be moved to the Results section as they described the participants (rather than the methods per se).

5. In the “Semi-structured Interviews” subsection, the authors mention interviews with 24 patients and 7 carers. However, Table 2 describes 36 patients and 7 carers. Could the authors comment on the difference in numbers reported?

6. Under Theme 1, the authors mention that “Cultural and socio-economic barriers were identified” but the said barriers are not described. I was left wanting more from the authors to better illustrate the cultural barriers. For example, the authors previously mention “housing, self-efficacy, and stigma of illness” as barriers that care teams attempted to address. Since ethnicity and cultural beliefs are highlighted as important source of variability in uptake of home dialysis throughout the manuscript, it would be helpful to provide a specific example of ethnic/cultural barrier identified (and/or a relevant quote on the subject). The authors later beautifully refer to the cultural characteristics of the care team, with a relevant quote to illustrate Theme 2 in this regard. An equivalent example for Theme 1 would be beneficial.

7. Under Theme 3, the authors mention “The inclusion of patient leaders and their role in the co-production of services”. The authors then describe training sessions and peer-support initiatives involving patients. Could the authors comment a little bit more on the involvement of the “patient leaders”? I think this additional information would be helpful to anyone looking to translate the findings from this study to their local program.

8. Discussion – Limitations subsection

“A limitation to generalisability is that all these sites are working within a healthcare system that that is free at the point of care” – The word “that” is repeated.

9. Footnote of Table 1: “As the time (…)” – should probably read as “At the time (…)”

10. Table 2 – “Multiple index of deprivation quartile” – This metric should be described in a footnote. Is this a score based on patients’ residence location/postal code?

11. Table 3 – Consider using subcategories in the table (e.g. “nurses” and then list all types of nurses described). Also, consider grouping some titles (e.g. “lead nurse”, “home therapies lead nurse”, “nurse manager”). Some job titles are not titles broadly used (e.g. “low clearance nurse specialist”) and may benefit from a description of the job title as a footnote.

VERSION 1 - AUTHOR RESPONSE

Reviewer 1 comments	Revisions to address comment
1.The abstract very well describes the aim of the study. However, the present study is only very briefly mentioned in the Introduction section and the aim is not mentioned clearly in the main text. The objective of the study should be added at the end of the introduction.	1.The last sentence of the introduction has been edited to more clearly state the study objective.
2."Sample : Site and participant selection" subsection – last sentence of 1st paragraph: "Selection of these four case study sites was designed to observe successful practice whilst maximising diversity, involved two-stage purposive sampling." – The authors might wish to add the word "and" before the word "involved" or modify "involved" for "involving".	2. 'and' added to improve readability. (Sample: Site and participant selection subsection – last sentence of 1st paragraph)
3. The authors discuss variability in uptake of home dialysis related to ethnicity in the introduction and designed their study to include centers with high uptake of home dialysis amongst ethnic minorities. Ethnicity of the participants is also described in Table 2. However, what "ethnic minorities" represent in the setting of the study is not described. Since ethnic minorities vary widely across regions/countries, it would be helpful to the readers to briefly describe the UK population and what constitutes "ethnic minorities".	In the UK kidney failure context this means Black, Asian and mixed race predominantly (as collected by the UK Renal Registry, using the classification system used by the Office of National Statistics: REF https://www.ons.gov.uk/methodology/classificationsandstandards/measuringequality/ethnicgroupnationalidentityandreligion:. Text has been added to the introduction (first paragraph) to clarify.<br>In table 2 Black and Asian are highlighted specifically to give the reader insight into what ethnicities have been included.
4. The sentences referring to Tables 2 and 3, as well as the first sentence from the "Semi-structured Interviews" subsection should maybe be moved to the Results section as they described the participants (rather than the methods per se).	We recognise presentation of qualitative research methods and results are different to what readers might be used to. However to represent this ethnography well the results of the qualitative analysis are presented in the results section. As ethnographers we view the participant samples as part of the research design, rather than being the result of the analysis.
5. In the "Semi-structured Interviews" subsection, the authors mention interviews with 24 patients and 7 carers. However, Table 2 describes 36 patients	The difference in numbers is because 24/36 patients were involved in interviews. Overall a total of 36 were involved in either the interview or the observations.

and 7 carers. Could the authors comment on the difference in numbers reported?	The following text has been added when table 2 is introduced to explain the difference. **Not all patients that were observed also took part in interviews, this explains the difference between the number of patient interviewees (24) and patients participating overall (36).**
6. Under Theme 1, the authors mention that "Cultural and socio-economic barriers were identified" but the said barriers are not described. I was left wanting more from the authors to better illustrate the cultural barriers. For example, the authors previously mention "housing, self-efficacy, and stigma of illness" as barriers that care teams attempted to address. Since ethnicity and cultural beliefs are highlighted as important source of variability in uptake of home dialysis throughout the manuscript, it would be helpful to provide a specific example of ethnic/cultural barrier identified (and/or a relevant quote on the subject). The authors later beautifully refer to the cultural characteristics of the care team, with a relevant quote to illustrate Theme 2 in this regard. An equivalent example for Theme 1 would be beneficial.	A quote and paragraph has been added at the end of theme 1 about how economic, cultural and individual factors are experienced simultaneously. One consultant observed the implications for their own practice i.e. addressing structural factors such as finance and housing as soon as possible to allow the patient the right conditions to engage with and build confidence for the treatment.
7. Under Theme 3, the authors mention "The inclusion of patient leaders and their role in the co-production of services". The authors then describe training sessions and peer-support initiatives involving patients. Could the authors comment a little bit more on the involvement of the "patient leaders"? I think this additional information would be helpful to anyone looking to translate the findings from this study to their local program.	Detail added to theme 3.
8. Discussion – Limitations subsection "A limitation to generalisability is that all these sites are working within a healthcare system that that is free at the point of care" – The word "that" is repeated.	removed
9. Footnote of Table 1: "As the time (...)" – should probably read as "At the time (...)"	Changed
10. Table 2 – "Multiple index of deprivation quartile" – This metric should be described	Added *** Score based on patients' home postcode to identify the level of deprivation of the area**

in a footnote. Is this a score based on patients' residence location/postal code?	**they live in, using the ranking system of the IMD (every area in England is ranked from 1 = most deprived, to 32,844 = least deprived), and converting this rank into deprivation quartile.**
11. Table 3 – Consider using subcategories in the table (e.g. "nurses" and then list all types of nurses described). Also, consider grouping some titles (e.g. "lead nurse", "home therapies lead nurse", "nurse manager"). Some job titles are not titles broadly used (e.g. "low clearance nurse specialist") and may benefit from a description of the job title as a footnote.	Nurses and allied health professionals added as subcategories.
**Reviewer 2 comments**	**Revisions to address comments**
1. I have some concerns with the novelty of the conclusions presented. Does it really surprise anyone that programs which are patient-centred, offer a menu of therapy options and have an administration that openly advocates for home therapies would, in turn, have higher uptake of home therapies? This is the subject of multiple narrative and systematic reviews over the decades. Is the novelty in that this is an ethnographic study? If so, is this simply using a different research approach to identify issues that are otherwise either self-evident or already known? To turn this around, wouldn't it be a surprise if one were NOT to have identified "transparency about all treatment options, minimizing assumptions about eligibility, working with people to overcome perceived barriers" as facilitators of home therapies uptake? Or more bluntly, to identify that limited transparency about treatment options were to lead to increased uptake? I do think that the authors would benefit from clearly articulating what about their approach and conclusions crystalizes something new.	1. We have articulated the novelty of the findings in the discussion section. To solve the stubborn problem of low home dialysis uptake, most have focussed on how services are organised rather than changing institutional culture. While this may seem obvious, it is not borne out in the way the problem has been approached to date.<br><br>Patient/person-centred practice tends to be poorly defined and ambiguous as a concept. Our study maps service activity back to the core organisational values and in doing so articulates what 'person-centred care' looks and feels like in this particular service context.<br><br>The following text was edited to make novel contribution clearer:<br>**'This 'focussed ethnography' is novel in its approach, highlighting aspects of organisational culture that are relevant to successful home dialysis practice, particularly concerning presentation of treatment choice and decision-making, for example by adopting the stance of 'presumption of eligibility'. Where previous studies have identified barriers and promising interventions, our findings also focus on the underlying organisational values which can assist the effective implementation of home dialysis innovation. These findings offer insights for leaders and managers directly responsible for shaping the organisational culture of kidney centres. It also informed the design of the quantitative components of the Inter-CEPt study such as**

	**the National Survey of Home Dialysis Centres in England by ensuring that questions related to organisational culture were included. The results of this survey corroborated the ethnography findings '**
2. The choice of centres to include: I find it curious to have included centres that only had a 9% or 13% prevalence proportion of home therapies rather than focusing on the one centre that has 23% uptake. The former sites would in many jurisdictions be considered poor performing sites and an interesting question might have been to explore the differences in the high performing site compared to those with less uptake. What makes the 23% site so much more successful than all the others? This study doesn't somehow stratify their results so see what the differences are between good sites and average sites. Are there elements to the three themes they have identified that are different in the 23% site versus the other three which are so clearly discordant? The way the study is framed now, there would be nothing unique to learn from the one site that actually does a very good job at attracting home therapies patients. This study hasn't leveraged the fact that one site is so much better than all the others to learn what that site does that is unique and makes them so successful. What are those characteristics? As it is, we are learning from average sites and mixing the findings with that of a good site? I am much more interested in what site 1 has to teach us about their culture compared to other institutional cultures – I wouldn't know that on the basis of the present results.	Selection of these four case study sites was designed to observe successful practice, whilst maximising diversity. In using a positive deviance approach it was important to capture success in the sample, but this wasn't the only consideration. Our sites were all performing above the national median for home dialysis uptake. However they also represented areas with different patient populations, including the capturing socio-economic and ethnic variation where we know inequities persist. We argue that just focusing on one site would have limited the generalisability and transferability of findings, although, we agree, it would make an interesting case study. We would also emphasise that the ethnographic study was utilised to inform the design of other components of the Inter-CEPt study, including the national survey, so it was very important to have a 'whole' country picture.<br><br>The analysis we used was thematic - an inductive process that involves repeated engagement with the data to allow themes to emerge across all sites. The value of thematic analysis here was to find patterns that apply to broader groups. We did not choose to perform comparative data analysis, however this would make an interesting alternative study.
3. The methods section makes a point to comment on the inclusion of sites with higher than typical uptake of home therapies among ethnic minorities but then this issue doesn't come up again at all. Was this intentional? Was there something to be learned specifically about programs with high ethnic diversity? If so, this doesn't come out at all later. If it	3.We have added a new quote in theme 1 which highlights the intersectionality of economic, cultural, personal factors and implications for practice.<br><br>The findings and discussion highlight housing, self-efficacy, stigma of illness as issues common to groups underrepresented in home therapies uptake (theme1).

doesn't matter, then why was this selection criteria important? Related to point (2) above, is there something about the culture of site 1 with respect to recruitment of ethnic minorities that allows this site to have twice the prevalence proportion of ethnic minorities compared to sites 2-4.	In theme 2 findings we cover the importance of staff cultural characteristics and language skills.<br><br>In the discussion, individualised decision-making support and issues 'access' are linked to underrepresented patients' needs.<br><br>Different sites had different patient populations, which makes measurement and comparison challenging. The study is not seeking to establish exact cause and effect for practices at each different site. It seeks to learn from positive and inclusive practice at all four sites.
4. Page 8 lines 13-29: I am unclear who are what is meant by "non-participant observation" – is this meant to describe review of physical locations (waiting rooms, clinical spaces, training areas, etc), promotional and educational material, etc? This is confusing because the section ends by stating that individual consent was obtained and that all involved were considered "participants in their own right."	4.Agree. This terminology of non-participant observation made sense in the initial protocol but doesn't read as well in this paper.<br><br>We have re-titled the section 'Observation'. We have clarified at the end of the section which observations we recorded consent for and which we didn't.
5. I find theme 3 to have a confusing title and isn't intuitive relative to the content of the paragraphs that follow or to the material presented in the table 4.	5. Text in table 4 adapted to make the link a clearer.<br><br>The paragraphs in 'Results' theme 3 outline how patient benefits are the driving motivator for treatment choices, as a key aspect of organisational culture. They go on to cover how the organisation work towards creating sustained change with other stakeholders.
